# Additional Fuel Allowances

**Published:** 1918-11-02

**Authors:** 


### Additional Fuel Allowances.

In response to an inquiry made by the Secretary of the National League for Health, Maternity, and Child Welfare, the Controller of Coal Mines states that a claim for an additional allowance of fuel under the Household Fuel and Lighting Order 1918 can certainly be submitted on the ground of illness in the case of childbirth, and would be allowed for a reasonable period thereafter. The maximum quantity of fuel that may be granted on such a claim is five tons, but the actual quantity granted must depend on the circumstances of each case.

